# Long-term clinical outcomes in critically ill patients with sepsis and pre-existing low muscle mass: a retrospective cohort study

**DOI:** 10.1186/s12871-023-02274-y

**Published:** 2023-09-15

**Authors:** Nola Darden, Sonakshi Sharma, Xue Wu, Benjamin Mancini, Kunal Karamchandani, Anthony S. Bonavia

**Affiliations:** 1 Department of Anesthesiology and Perioperative Medicine, Penn State Milton S Hershey Medical Center, 500 University Dr, Mailbox H-187, Hershey, PA 17033 USA; 2 Penn State College of Medicine, Hershey, PA USA; 3 Department of Public Health Sciences, Penn State College of Medicine, Hershey, PA USA; 4 Department of Anesthesiology and Pain Management, University of Texas Southwestern Medical Center, Dallas, TX USA; 5 Division of Critical Care Medicine, Department of Anesthesiology and Perioperative Medicine, Penn State Milton S Hershey Medical Center, 500 University Dr, Mailbox H-187, Hershey, PA 17033 USA

**Keywords:** Sarcopenia, Low muscle mass, Critical illness, Sepsis, Prognostication, Long-term outcomes

## Abstract

**Purpose:**

Critically ill patients with sepsis account for significant disease morbidity and healthcare costs. Low muscle mass has been proposed as an independent risk factor for poor short-term outcomes, although its effect on long-term outcomes remains unclear.

**Methods:**

Retrospective cohort analysis of patients treated at a quaternary care medical center over 6 years (09/2014 - 12/2020). Critically ill patients meeting Sepsis-3 criteria were included, with low muscle mass defined by $\le$ 5^th^ percentile skeletal muscle index, measured at the L3 lumbar level (L3SMI) on Computed-Tomography (CT) scan ($\le$ 41.6 cm^2^/m^2^ for males and $\le$ 32.0 cm^2^/m^2^ for females). L3SMI was calculated by normalizing the CT-measured skeletal muscle area to the square of the patient’s height (in meters). Measurements were taken from abdominal/pelvic CT scan obtained within 7 days of sepsis onset. The prevalence of low muscle mass and its association with clinical outcomes, including in-hospital and one-year mortality, and post-hospitalization discharge disposition in survivors, was analyzed. Unfavorable post-hospitalization disposition was defined as discharge to a location other than the patient’s home.

**Results:**

Low muscle mass was present in 34 (23%) of 150 patients, with mean skeletal muscle indices of 28.0 ± 2.9 cm^2^/m^2^ and 36.8 ± 3.3 cm^2^/m^2^ in females and males, respectively. While low muscle mass was not a significant risk factor for in-hospital mortality (hazard ratio 1.33; 95% CI 0.64 – 2.76; *p* = 0.437), it significantly increased one-year mortality after adjusting for age and illness severity using Cox multivariate regression (hazard ratio 1.9; 95% CI 1.1 – 3.2; *p* = 0.014). Unfavorable post-hospitalization discharge disposition was not associated with low muscle mass, after adjusting for age and illness severity in a single, multivariate model.

**Conclusion:**

Low muscle mass independently predicts one-year mortality but is not associated with in-hospital mortality or unfavorable hospital discharge disposition in critically ill patients with sepsis.

## Introduction

Sepsis is defined as the life-threatening organ dysfunction that is caused by a dysregulated host response to infection [1]. Particularly adverse clinical outcomes are observed in septic patients who are older than 65 years [2], although advances in critical care therapy have allowed these patients to live longer and experience significant, previously unseen morbidity. The frequent coexistence of sarcopenia and sepsis in older patients has created a need to understand how these conditions interact to influence patient outcomes [3–5]. This knowledge may allow clinicians to plan for appropriate rehabilitation and nursing care, early in course of hospitalization in high-risk patients. It may also facilitate discussions by clinicians with patients and their families regarding anticipated disease prognosis and long-term goals of medical care.

Sarcopenia is a syndrome defined by both loss of muscle mass and muscle function [6, 7], the latter being challenging to establish in critically ill patients. Skeletal muscle index, measured at the L3 lumber level of Computer-Tomography images (L3SMI), has therefore been described as a surrogate for sarcopenia [8]. A recent, prospective study of 187 patients confirmed that a low L3SMI significantly correlated with age and mid-arm muscle circumference among males, and was associated with poor survival even after adjusting for age and sex [9]. The use of Computer-Tomography (CT) images to quantify total body skeletal muscle dates back almost two decades [10], although other measures of low muscle mass are being increasingly employed today. Dual energy X-ray absorptiometry [11], magnetic resonance imaging [12], ultrasound [13], and bioelectrical impedance analysis [14] can also be employed for measuring muscle mass. However, accurate measurements of muscle mass together with the wide availability and rapidity of CT imaging makes it particularly well-suited to the care of critically ill patients, who often cannot tolerate prolonged testing.

The first study that investigated the role of muscle mass in septic patients was conducted in 2017, reporting an increased in-hospital mortality in older patients with sepsis and low muscle mass [15]. Several studies conducted since then have been limited by short intervals to patient follow-up [16], as evidenced by a meta-analysis of post-sepsis outcomes in 2396 patients that reported early (in-hospital or 1-month) mortality as the primary measured outcome [17]. This limitation is not insignificant, since much of the disease morbidity related to sepsis occurs in the weeks to months following acute illness [18].

The importance of investigating sarcopenia as an independent risk factor for poor post-septic outcomes is underlined by aging global populations and the resultant demographic profile of those patients who are increasingly seeking healthcare [19]. In the present study, we hypothesized that low muscle mass would adversely predict *long-term* outcomes in critically ill patients with sepsis. To investigate our hypothesis, we performed a retrospective analysis of critically ill patients meeting Sepsis-3 criteria [1]. Besides one-year mortality as the primary outcome, we investigated the effect of low muscle mass on in-hospital mortality and unfavorable post-hospitalization discharge disposition. We used the latter as a surrogate marker of quality-of-life following patients’ index hospitalization. Specifically, unfavorable discharge disposition was defined as hospital transfer to a long-term rehabilitation or nursing facility, rather than home.

## Methods

### Study design

This was a single-center, retrospective cohort analysis of patients hospitalized at Penn State Milton S. Hershey Medical Center, a quaternary care medical center, between 09/2014 and 12/2020. Adults aged 18 years and older, who were identified as critically ill based the use of Centers for Medicare and Medicaid Services, Current Procedural Terminology (CPT) codes of 99291 or 99292, and who were diagnosed with sepsis according to the International Classification of Diseases, Ninth Edition, Clinical Modification (ICD-9-CM) sepsis codes were electronically screened for potential inclusion in the study. Patients who did not have a Computed-Tomography (CT) scan of their abdomen performed within 7 days of index hospitalization were then excluded. Given the known limitations of using administrative data such as *International Classification of Diseases, Ninth Edition, Clinical Modification* (ICD-9-CM) sepsis codes for identifying actual incidences of this disease [20, 21], research investigators manually ascertained which of the remaining patients met Sepsis-3 criteria [1]. A diagnosis of sepsis required that a patient experienced a change in sequential organ failure assessment (SOFA) score of two or more, in the setting of clinically suspected or microbiologically proven infection, in accordance with the Sepsis-3 criteria [1].

Demographic data, medical comorbidities and short- and long-term clinical outcomes were obtained from the electronic medical record. Medical comorbidities were identified using ICD-9 billing codes. Severity of acute illness was defined by Acute Physiology and Chronic Health Evaluation (APACHE) II and Sequential Organ Failure Assessment (SOFA) scores [22, 23], which were calculated by the research investigators. The manuscript was written according to the Strengthening the Reporting of Observational studies in Epidemiology (STROBE) criteria [24].

### Assessment of low muscle mass

**Fig. 1:**
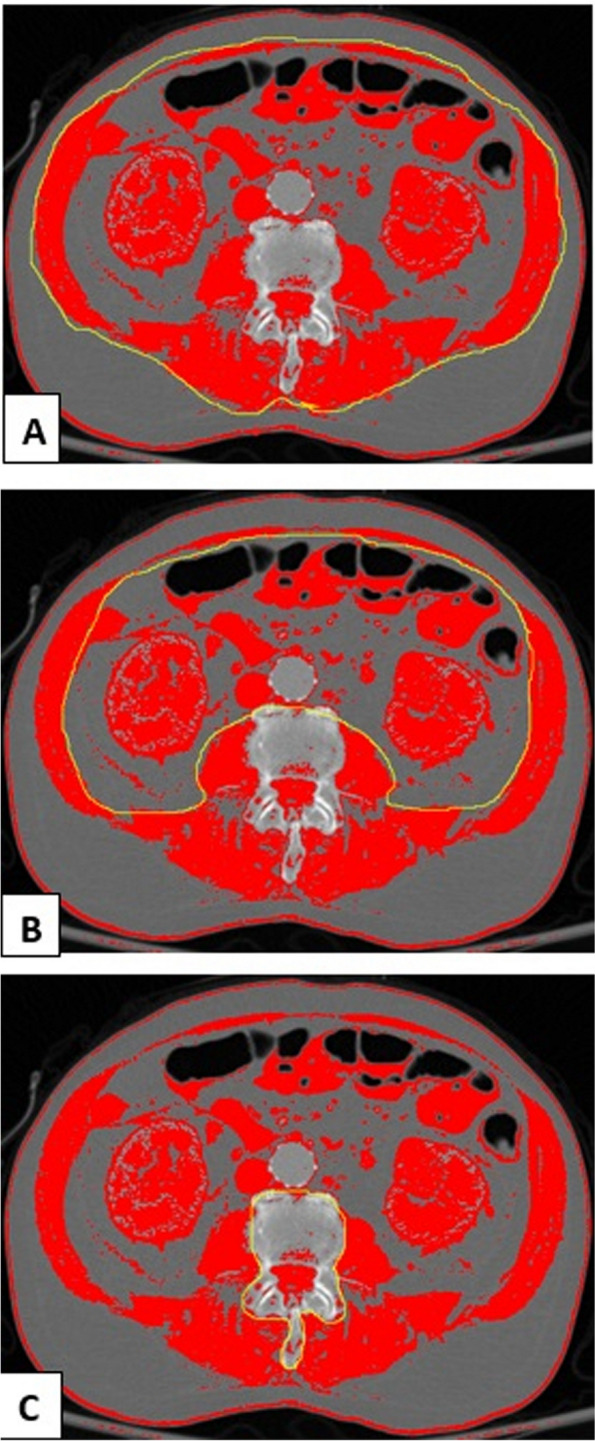
Measuring skeletal muscle area on L3 axial slice Computer-Tomography (CT) Image, Using the National Institutes of Health ImageJ. Radio density thresholds were selected to correlate with those of skeletal muscle, with lower and upper thresholds of − 29 and 150 Hounsfield units, respectively. **A** Outer perimeter of the abdominal muscles is first delineated (yellow tracing) and area calculated, **B** The process is repeated for the inner perimeter of abdominal muscles (yellow tracing), **C** Vertebral area is further excluded (yellow tracing). The difference between measures equates to the area of skeletal muscle at the L3 level

Axial CT scan images, 3.0 mm in thickness and taken at the L3 vertebral level were obtained from the Picture Archiving and Communication System (PACS), in Digital Imaging and Communication in Medicine (DICOM) format. Abdominal and/or pelvic CT imaging performed within 7 days of the date of index hospitalization were used to define low muscle mass. Specifically, low muscle mass was defined as $\le$ 5^th^ percentile of skeletal muscle index of a sex-matched, reference population (41.6 cm^2^/m^2^ for males and 32.0 cm^2^/m^2^ for females), as previously described [16, 25]. Skeletal muscle area was measured manually by using the freely-available ImageJ software (version 1.53t, U. S. National Institutes of Health, Bethesda, Maryland, USA) [26], as illustrated in Fig. 1 and using methods previously described [27]. Both CT images obtained with and without intravenous contrast were used in the calculation of skeletal muscle area, as previously reported [28]. Since muscle mass is related to age, we also analyzed the age-adjusted effects of low muscle mass on clinical outcomes. We used an age cutoff of 70 years to define ‘older’ versus ‘younger’ patients with sepsis, based on data from previous epidemiologic studies [2, 29, 30].

### Clinical outcomes

The primary outcome of one-year mortality was defined from the time of sepsis diagnosis during patients’ index hospitalization. In-hospital mortality, a secondary outcome, was defined as the occurrence of death during patients’ index hospitalization. ‘Favorable’ discharge disposition was defined as transfer of sepsis survivors from hospital directly to their home at the end of their index hospitalization, while ‘unfavorable’ discharge disposition was defined as transfer from hospital to a rehabilitation facility (skilled nursing facility or inpatient rehabilitation facility) or hospice care. All follow-up data for clinical outcomes was obtained from the electronic medical record > 18 months following the date of hospitalization of the latest septic patient included in the cohort, to allow sufficient time for mortality data to be reflected in the electronic medical record.

### Statistical analysis

Our sample size of 150 patients was based on two recent, comparable, retrospective cohort studies that assessed the effects of low muscle mass on short-term, clinical outcomes [15, 16]. For binary and nominal outcomes, we constructed frequencies and percentages as descriptive statistics, and implemented Fisher’s exact test statistics to compare patients with and without low muscle mass. For continuous outcomes, we constructed median and inter-quartile ranges (IQR) as descriptive statistics and implemented Wilcoxon rank-sum tests to compare groups with and without low muscle mass. One-year survival was defined as death within 365 days of discharge from index hospitalization and was analyzed via Kaplan–Meier survival curves and log-rank tests. In-hospital survival was similarly analyzed using survival status at hospital discharge. Cox regression was used to estimate the effect of muscle mass on in-hospital and one-year survival, correcting for SOFA and APACHE II illness severity scores. For this Cox regression, the proportional hazard assumption was assessed and found to be satisfied. Multivariable logistic regression was used to analyze the effect of muscle mass on the binary outcome of discharge disposition, adjusted for illness severity. The assumptions of logistic regression were satisfied as the observations were independent of each other and variables were found not to be too highly correlated with each other. Analyses were performed using R v4.1.2 (R core team, 2022). In all statistical analyses, the significance level was set at *p* = 0.05. Results with a *p* value less than or equal to 0.05 were considered statistically significant.

## Results

### Study population

**Fig. 2:**
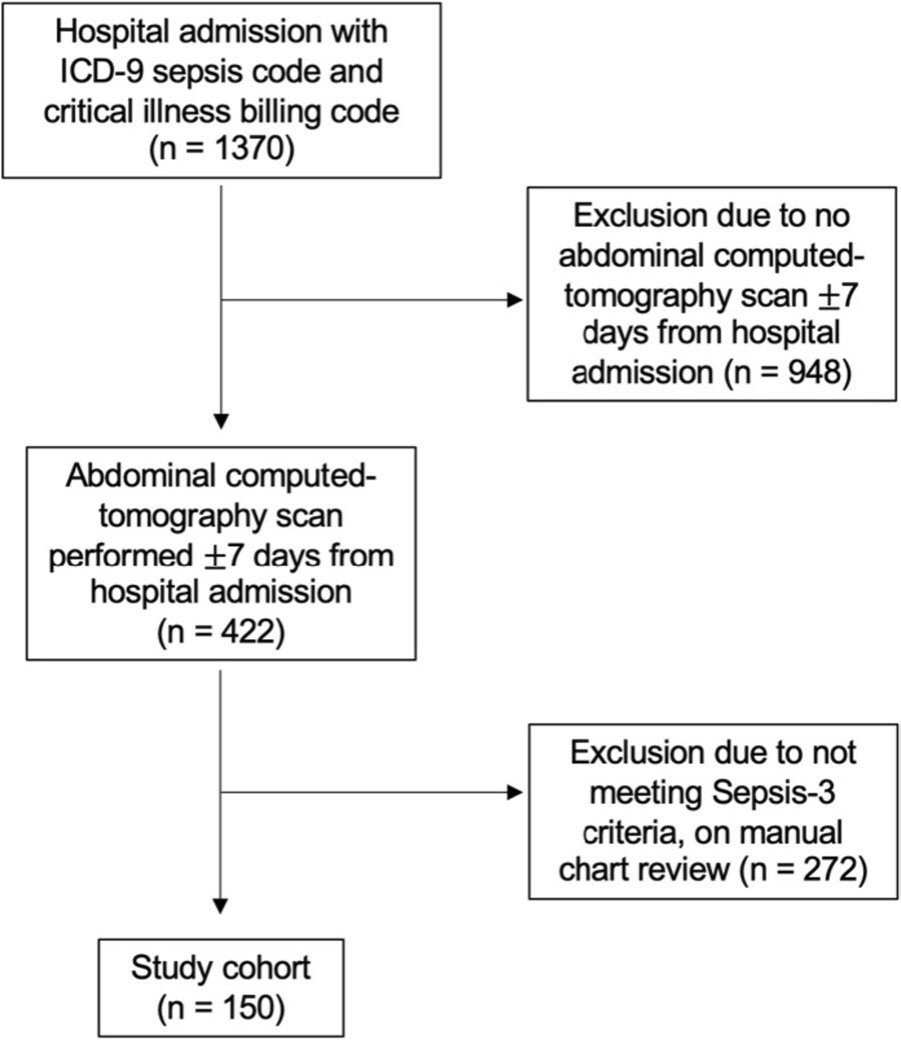
Flow diagram demonstrating numbers of individuals included and excluded at each stage of the study

Of the 150 patients that were included, 34 patients (23%) met criteria for pre-existing low muscle mass (Fig. 2). Age, SOFA and APACHE II scores for patients with low muscle mass were higher than those having preserved muscle mass (Table 1). Sex- and age-specific body composition parameters are shown Table 2.

**Table 1:** Demographic and clinical profile

	**Low muscle mass (*n* = 34)**	**Preserved muscle mass (*n* = 116)**	***P*-value**
**Patient Characteristics** (median, IQR)			
Age	75 (63,83)	63 (52,69)	**< 0.001**
Female	11 (32.4%)	52(44.8%)	0.238
BMI (kg/m^2^)	24 (21, 29)	29 (25,34)	**< 0.001**
Height (cm)	174 (166, 182)	170 (161,177)	0.062
Weight (kg)	74 (59, 88)	87 (70,99)	**0.032**
Race			0.270
Asian	1 (2.9%)	0	
Black or African American	2 (5.9%)	9 (7.8%)	
Two or More Races	0	3 (2.6%)	
White	31 (91.2%)	95 (81.9%)	
**Medical Co-morbidities**			
Diabetes mellitus	11 (32.4%)	39(33.6%)	1
Heart failure	6 (17.6%)	21(18.1%)	1
Kidney transplant	0	1 (0.9%)	
End-stage kidney disease	0	4 (3.4%)	
On hemodialysis	0	0	
**Illness severity (median, IQR)**			
SOFA	7 (4, 10)	5 (4, 7)	**0.025**
APACHE II	19 (14, 25)	15 (11, 21)	**0.019**
**Skeletal muscle area, cm**^**2**^** (mean, SD)**			
Males	116.2 ± 13.6	159.0 ± 20.8	**< 0.0001**
Females	74.5 ± 9.3	115.4 ± 21.6	**< 0.0001**
**Skeletal muscle index, cm**^**2**^**/m**^**2**^** (mean, SD)**			
Males	36.8 ± 3.3	51.3 ± 7.1	**< 0.0001**
Females	28.0 ± 2.9	44.8 ± 7.3	**< 0.0001**

**Table 2:** Body composition of critically ill patients with sepsis

Sex	Age (years)	n (% of sex)	L3-SMA (cm^2^)	L3-SMI (cm^2^/m^2^)
All males and females	-	150	131.1 ± 32.7	45.1 ± 9.5
All males	-	87 (100%)	147.7 ± 26.9	47.5 ± 9.0
Male	< 60	26 (30.0%)	157.2 ± 29.5	49.7 ± 9.2
Male	60–70	37 (42.5%)	153.3 ± 23.3	49.4 ± 8.9
Male	> 70	24 (27.6%)	128.7 ± 19.5	41.9 ± 6.5
All females	-	63 (100%)	108.2 ± 25.3	41.9 ± 9.3
Female	< 60	26 (41.3%)	117.0 ± 22.8	44.3 ± 8.4
Female	60–70	20 (31.7%)	108.3 ± 29.4	43.2 ± 10.1
Female	> 70	17 (27.0%)	94.7 ± 18.2	36.7 ± 8.1

Data are presented as mean (± SD)

*SMA* Skeletal Muscle Area, *SMI* Skeletal Muscle Index

### Association between low muscle mass and one-year survival

**Fig. 3:**
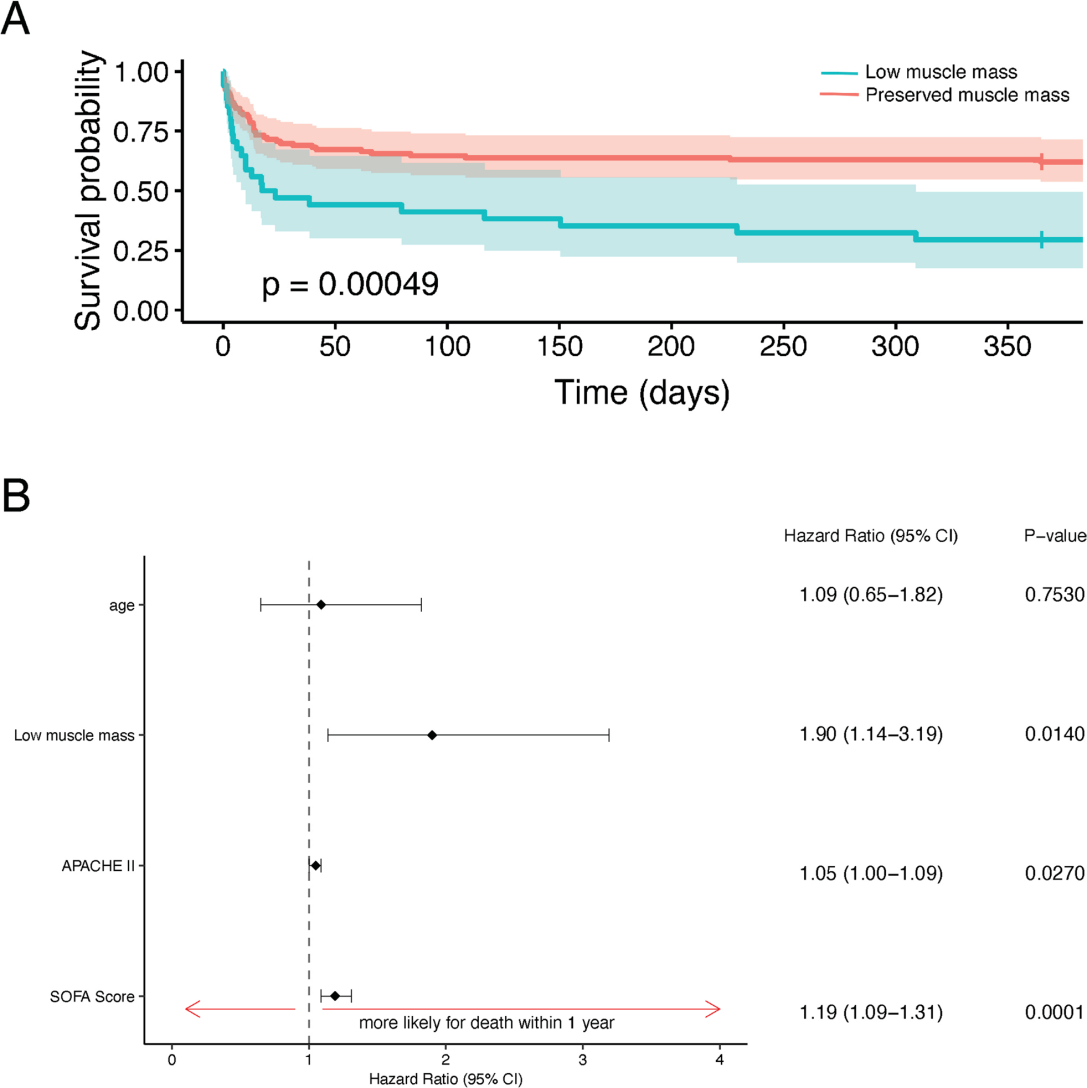
Association Between Low Muscle Mass and One-year Mortality. **A** Kaplan–Meier survival curves demonstrating the effect of low muscle mass on one-year mortality, in septic patients. 95% confidence intervals are shown. **B** Cox multivariable regression assessing the effect of low muscle mass on one-year mortality when adjusting for age and severity of illness by SOFA and APACHE II scores

Table 3 presents the results of the univariable analysis of clinical outcomes in patients having low and preserved muscle mass. In univariate analysis, low muscle mass significantly decreased one-year survival, with an estimated survival probability at one-year of 29.4% (95% CI: 17.5%-49.5%) in these patients, versus 62% (95% CI: 54%-72%) in patients with preserved muscle mass (*p* = 0.0005). One-year survival probability for patients with low versus preserved muscle mass is shown in Fig. 3A (hazard ratio = 2.4, 95% CI: 1.44–3.9, *p* < 0.001). After adjusting for age, APACHE II and SOFA score by Cox multivariable regression, low muscle mass remained an independent risk factor for one-year mortality (hazard ratio 1.9; 95% CI 1.1 – 3.2; *p* = 0.014) (Fig. 3B). For each one-unit increase in APACHE II, the risk of death within one year increased by a factor of 1.05 (95% CI: 1.00 – 1.09, *p* = 0.027). Similarly, for each one-unit increase in SOFA score, the risk of death within one year increased by a factor of 1.19 (95% CI: 1.09 – 1.31, *p* < 0.001). However, age did not emerge as a significant risk factor for the one-year survival outcome (Fig. 3B).

**Table 3:** Clinical outcomes in patients with and without low muscle mass

	**Low muscle mass (*n* = 34)**	**Preserved muscle mass (*n* = 116)**	***P*-value**
**Short-term outcomes**			
In-hospital mortality	13 (38%)	27 (23%)	0.121
**Post-hospitalization discharge disposition in survivors**			**< 0.001 **
Home	7 (21%)	56 (48%)	
Hospice	5 (15%)	8 (7%)	
Skilled nursing facility	8 (24%)	5 (4%)	
Inpatient rehabilitation facility	1 (3%)	18 (16%)	
Long-term care hospital	0	2 (2%)	
**One-year survival** (median, IQR)			
Survival time (days)	20 (4.2, 365)	365 (14, 365)	**0.003**
Deceased at 1-year follow up	24 (71%)	44 (38%)	**< 0.001 **
One-year survival probability (95% CI)	29% (18%—50%)	62% (54%—72%)	**< 0.001 **
Hazard ratio (95% CI)	2.4 (1.44, 3.9)		**< 0.001 **
Length of intensive care treatment (days)	1 (0.7, 2.0)	1 (0.1, 1.7)	0.348
Hospital Length of stay	6 (4, 10)	7 (4, 7)	0.736
**Hospital Readmission** (median, IQR)			
Patients readmitted within 1 year of hospital discharge	2 (6%)	20 (17%)	0.165
Days from discharge to readmission	5 (3, 8)	9 (4, 65)	0.424
Length of hospital readmission	6 (3, 9)	4 (2, 7)	1.000

### Association between low muscle mass and in-hospital mortality

**Fig. 4:**
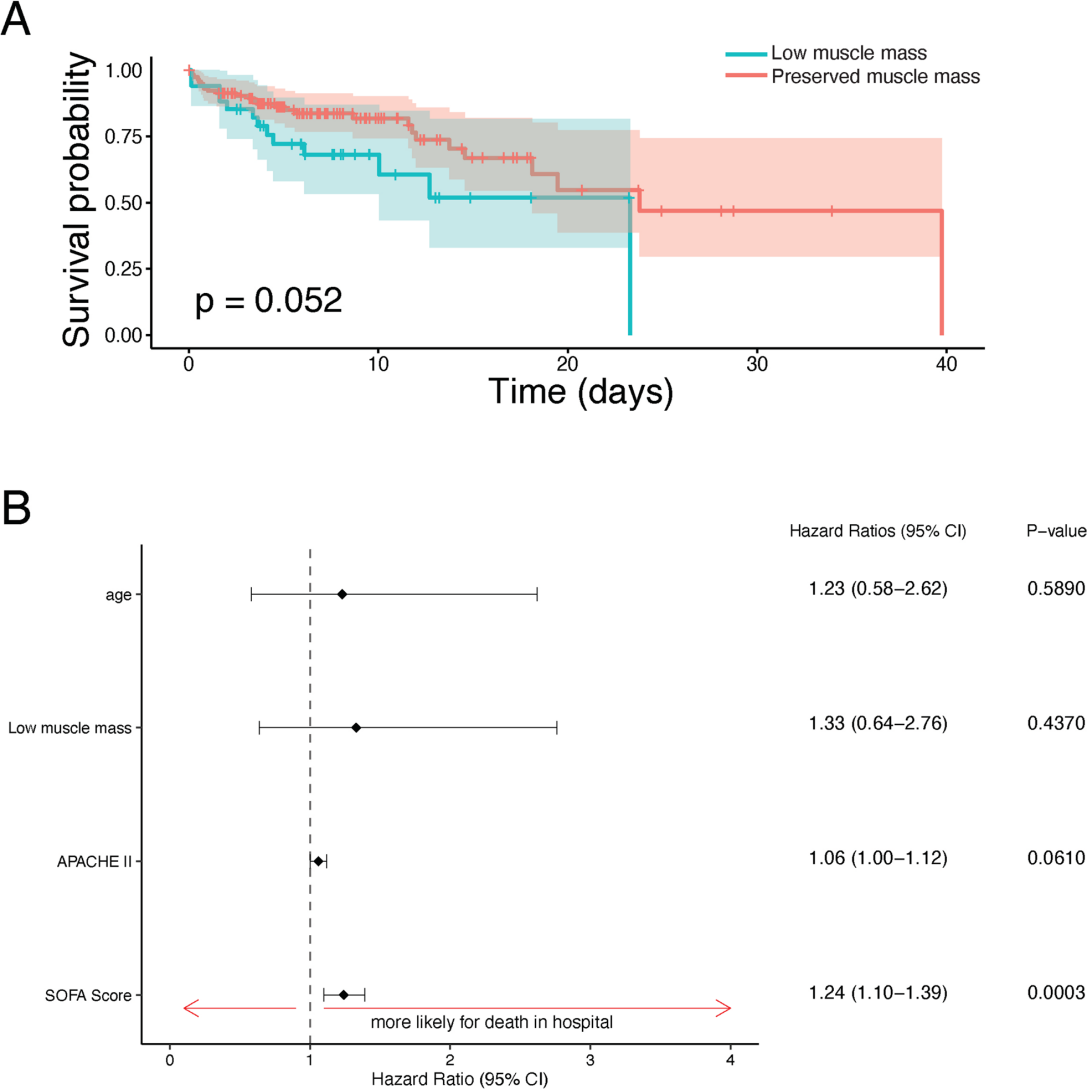
Association Between Low Muscle Mass and In-hospital Mortality. **A** Kaplan–Meier survival curves demonstrating the effect of low muscle mass on in-hospital mortality, in septic patients. 95% confidence intervals are shown. **B** Cox multivariable regression assessing the effect of low muscle mass on in-hospital mortality when adjusting for age and severity of illness by SOFA and APACHE II scores

In univariable analysis, low muscle mass non-significantly decreased in-hospital survival (hazard ratio 1.93; 95% CI: 0.98 – 3.78; *p* = 0.056). In-hospital survival probability for patients with low versus preserved muscle mass is shown in Fig. 4A. After adjusting for age, SOFA score and APACHE II by Cox multivariable regression, low muscle mass was not a significant risk factor for in-hospital mortality (hazard ratio 1.33; 95% CI 0.64 – 2.76; *p* = 0.437) (Fig. 4B). For each one-unit increase in APACHE II, the risk of death in the hospital shows a trend towards an increase by a factor of 1.06, although this did not reach statistical significance (95% CI: 0.997 – 1.12, *p* = 0.061). For each one-unit increase in SOFA score, the risk of death in the hospital increased significantly by a factor of 1.236 (95% CI: 1.10 – 1.39, *p* < 0.001). Age did not emerge as a significant risk factor for in-hospital mortality (Fig. 4B).

### Association between low muscle mass and hospital discharge disposition

**Fig. 5:**
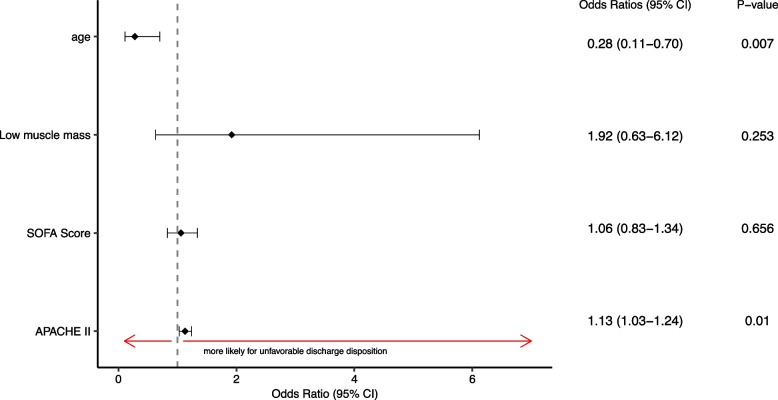
Logistic analysis assessing the effect of low muscle mass on discharge disposition when adjusting for age and severity of illness by SOFA and APACHE II score

Univariable logistic regression analysis revealed that the proportion of patients experiencing unfavorable post-hospital discharge disposition was higher in patients having low muscle mass, affecting 67% of these patients (odds ratio 3.4; 95% CI: 1.3 – 9.8; *p* = 0.017) (Table 4). However, in multivariate logistic regression adjusted for age, APACHE II and SOFA score, the odds of having an unfavorable hospital discharge were not significant in patients having low muscle mass (odds ratio 1.92, 95% CI: 0.63 - 6.12, *p* = 0.253). Younger septic patients were less likely to experience unfavorable disposition compared to older patients, by a factor of 0.3 (95% CI: 0.11 - 0.70, *p* = 0.007). Patients with higher APACHE II score also had an increased likelihood of an unfavorable discharge disposition (OR = 1.1, 95% CI: 1.03 –1.24, *p* = 0.01) (Fig. 5).

**Table 4:** Univariable logistic analysis of the effect of low muscle mass on hospital discharge disposition

	**Low muscle mass (*n* = 21)**	**Preserved muscle mass (*n* = 89)**	**Odds Ratio (95% CI)**	***P*-value**
Favorable post-hospitalization discharge	7 (33%)	56 (63%)	3.4 (1.3 – 9.8)	**0.017 **
Unfavorable post-hospitalization discharge	14 (67%)	33 (37%)

## Discussion

Rapid and accurate assessments of sarcopenia have gained increasing traction amongst clinicians seeking to risk-stratify patients requiring medical care [31, 32]. In the present analysis, we used low muscle mass as a surrogate for sarcopenia, with numerical cut-offs for low muscle mass that were derived from a race-matched population described in a prior investigation [16]. We observed a 23% prevalence of pre-existing low muscle mass in critically ill patients with sepsis, as compared with a reported prevalence ranging between 5 and 31% [15, 16]. We also identified low muscle mass as an independent risk factor for one-year mortality, although it did not increase the risk for in-hospital mortality or for unfavorable hospital discharge disposition.

While our investigation did not find an association between low muscle mass and short-term mortality following sepsis, Oh et al. reported both an increase in short- and long-term mortality risk, in a single-center, retrospective study of patients with septic shock and sarcopenia (defined by abdominal muscle area on CT scan) [33]. However, it is important to note that this study only included patients with shock, who inherently experience higher mortality rates as compared with patients having sepsis alone [34]. Additionally, the study by Oh et al. did not evaluate the impact of sarcopenia on post-hospitalization discharge status, which could serve as a surrogate measure for quality of life in survivors of critical illness. Our findings align more closely with those reported in a prospective, single-center investigation of 47 sepsis patients, concluding that pre-existing sarcopenia is independently associated with poor long-term functional status and increased one-year mortality [35]. Interestingly, the latter study demonstrated that, while sepsis-induced critical illness leads to an acute and persistent loss of muscle mass at three months, it does not have the same effect on long-term functional status and one-year mortality as pre-existing sarcopenia does [35].

The juxtaposed findings of unchanged in-hospital mortality and increased one-year mortality, in critically ill patients with sepsis and pre-existing low muscle mass, is notable. It is plausible that patients with significant medical comorbidities, including low muscle mass, survive their index hospitalization but are then more likely to require the intensive support provided by skilled nursing services and rehabilitation facilities following discharge from the hospital. One-year mortality data may provide more objective insight into the long-term effects of muscle dysfunction in patients who survive hospitalization but continue to experience high post-hospitalization care needs. Further prospective research is needed to discern the influences of low muscle mass on post-hospitalization care.

Our investigation does not allow us to differentiate between acute loss of muscle mass due to sepsis and other factors affecting critically ill patients [36], and chronic loss of muscle mass. However, it is essential to consider that skeletal muscle plays a significant role in the influence of body composition on clinical outcomes, as it governs crucial functions like mobility, strength, and balance [37]. Several studies have shown a clear association between reduced muscle mass and unfavorable outcomes, including decreased ventilator-free and ICU days [38] and shorter overall survival [39, 40]. Moreover, sepsis induces hypermetabolism and inflammation, leading to protein breakdown, which is further exacerbated by inadequate nutrition [41]. The immobility resulting from critical illness also contributes to muscle disuse and atrophy [42].

The combination of low muscle mass and sepsis is likely to produce a synergistic negative effect in critically ill patients. While the retrospective nature of our investigation precludes us from determining causal effects, it is plausible that low muscle mass acts as both a causative factor and as an outcome associated with a poor long-term prognosis. When low muscle mass is identified in a patient, clinicians can take several steps to intervene and address this issue. Assuming that sepsis is being appropriately managed, nutritional support is of utmost importance for maintaining and building muscle mass [43]. Physical therapy and exercise, including early mobility and ambulation, are also particularly important and supported by an extensive body of literature that associates these interventions with improved clinical outcomes [44]. Respiratory therapy and protocolized daily spontaneous awakening and breathing trials, in mechanically ventilated patients, may lead to shorter duration of mechanical ventilation and earlier mobility, thus preserving muscle mass [45, 46]. Collaborative care amongst a multidisciplinary care team of physicians, nurses, dieticians, and physical therapists can provide comprehensive care in a challenging patient population, thus improving outcomes.

Unlike frailty assessments, which require an interview of patients or their families to assess slowness, weakness, weight loss and exhaustion [47, 48], image-based assessments of muscle mass do not require reliable information about one’s past medical history. This is a crucial advantage when caring for critically ill patients, since these patients often suffer from confusion and/or encephalopathy and are often admitted emergently to the hospital in the absence of family members who could corroborate their medical history. Body mass index (BMI) provides an objective measure of body proportions, and it has been associated with long-term prognosis including mortality [49]. However, BMI does not differentiate between weight derived from fat, muscle, bone, or water. An elevated BMI could result from either a high body fat content or significant muscle mass. Assessments like SMA/SMI offer a more nuanced understanding of an individual's body composition. CT imaging-based risk assessment tools are also particularly useful in sepsis since these patients often undergo imaging as part of their medical work-up and the results are rapidly available within the electronic medical record.

Strengths of our study include a complete data set of one-year outcomes for all patients, and the validation of sepsis diagnoses by research investigators, according to contemporary Sepsis-3 criteria. Its main limitations are its retrospective nature, its predominantly Caucasian population (reflective of the healthcare system’s catchment area), and its single-center design. Selection bias may have been introduced by the inadvertent exclusion of patients that did not undergo an abdominal CT scan because the origin of sepsis was clearly of non-abdominal origin. While in-hospital mortality was not different between septic patients with and without low muscle mass, the former group did experience significantly shorter median survival times, which could potentially affect statistical comparison of one-year mortality rates. Furthermore, comorbidities were primarily based on ICD-9 codes (potential underreporting bias) and critical illness was defined by billing information provided by the physician. The latter definition of critical illness would not necessarily mean that a patient was admitted to an intensive care unit for sepsis care, as a physician can also bill for critical care therapy in an emergency department setting. The inclusion of critically ill patients with sepsis, a population that experiences high mortality rates and frequent discharge to a rehabilitation facility, may also necessarily limit the external validity of our results. However, this population accounts for a large proportion of the morbidity and healthcare costs, and it is therefore of interest to epidemiologists, physicians, and healthcare administrators alike [50].

## Conclusions

Low muscle mass, as indicated by a reduced L3SMI measured on abdominal CT within 7 days of sepsis onset, is independently associated with increased one-year mortality but is not associated with hospital mortality or unfavorable discharge disposition. These findings have significant implications for clinical management, research, and quality of life in patients with sepsis. For clinicians, regular assessment of muscle mass, possibly through L3SMI measurements, could become a routine aspect of sepsis patient evaluations. Identifying at-risk patients early may facilitate targeted interventions, such as nutritional support or physical therapy, to counteract muscle loss and potentially improve long-term outcomes. Given the demonstrated association between low muscle mass and one-year mortality, future research should delve deeper into understanding the mechanisms behind this correlation. Moreover, intervention studies could be designed to explore ways of preserving or increasing muscle mass in sepsis patients and to evaluate their impact on both short-term and long-term outcomes. Regarding patient function, a deeper comprehension of the epidemiology and clinical consequences of low muscle mass can guide strategies to enhance both survival rates and the functional well-being of sepsis survivors. By highlighting the potential repercussions of muscle atrophy in critically ill sepsis patients, this study underscores the need for multi-disciplinary approaches that go beyond immediate clinical management, encompassing aspects of rehabilitation, nutritional guidance, and long-term patient care.

## Data Availability

All data resulting from this analysis is contained within the manuscript.
